# Applying the latest advances in genomics and phenomics for trait discovery in polyploid wheat

**DOI:** 10.1111/tpj.14150

**Published:** 2018-12-19

**Authors:** Philippa Borrill, Sophie A. Harrington, Cristobal Uauy

**Affiliations:** ^1^ School of Biosciences The University of Birmingham Birmingham B15 2TT UK; ^2^ John Innes Centre Norwich Research Park Norwich NR4 7UH UK

**Keywords:** genetics, phenotyping, crop improvement, *Triticum aestivum*, natural variation, homoeolog, polyploidy, genetic diversity, gene validation

## Abstract

Improving traits in wheat has historically been challenging due to its large and polyploid genome, limited genetic diversity and in‐field phenotyping constraints. However, within recent years many of these barriers have been lowered. The availability of a chromosome‐level assembly of the wheat genome now facilitates a step‐change in wheat genetics and provides a common platform for resources, including variation data, gene expression data and genetic markers. The development of sequenced mutant populations and gene‐editing techniques now enables the rapid assessment of gene function in wheat directly. The ability to alter gene function in a targeted manner will unmask the effects of homoeolog redundancy and allow the hidden potential of this polyploid genome to be discovered. New techniques to identify and exploit the genetic diversity within wheat wild relatives now enable wheat breeders to take advantage of these additional sources of variation to address challenges facing food production. Finally, advances in phenomics have unlocked rapid screening of populations for many traits of interest both in greenhouses and in the field. Looking forwards, integrating diverse data types, including genomic, epigenetic and phenomics data, will take advantage of big data approaches including machine learning to understand trait biology in wheat in unprecedented detail.

## Significance Statement

The objective of this review was to discuss how the latest developments in genomics and phenomics can be applied to understand the biology of agronomic traits in polyploid wheat. We describe the new resources and technological developments in the field, and discuss how they can be integrated to improve gene discovery and validation in wheat.

## Introduction

Crop production must increase to meet the food, feed and fuel demands of a global population estimated to exceed nine billion by 2050 (UN, 2015). Currently, one in nine people live under food insecurity (FAO, 2015). With limited opportunity to expand agriculture on existing land, increasing yields could significantly reduce the number of people at risk of hunger (Rosegrant *et al*., 2013). Despite the need for a 50% increase in crop production by 2050 (Tilman *et al*., 2011), our current rates of yield increase are insufficient to reach this goal (Ray *et al*., 2013). It is therefore critical and urgent that we identify approaches to increase crop productivity, for example through genetically improving cultivars (see Box 1 for glossary of terminology) and improving agronomic practises (Spiertz, 2012; Hawkesford *et al*., 2013).

Here we focus on polyploid wheat, which accounts for more than 20% of the protein and caloric intake of humans. Compared with other major (diploid) cereals, for example rice and maize, wheat has lagged in the development of genomic resources and the genetic understanding for many major productivity traits (e.g. yield, abiotic stress tolerance). This lag was principally due to its polyploid genome, which makes genetic analyses in wheat more cumbersome than in diploids (Bevan *et al*., 2017) and is often a barrier for researchers to understand the biology directly underlying traits in wheat (Box 1). The fact that many productivity traits are inherited in a quantitative manner and require field‐based phenotyping also compounds the issues. However, over the past few years, many of the entry barriers to wheat research have been dramatically lowered thanks to multiple genomics developments and the availability of open access resources (Uauy, 2017). For many purposes, wheat can now be treated (almost) like a model crop species for trait discovery.

Box 1 **Glossary of terminology**

**Table:** 

Term	Definition
Breeding programme	The development of novel plant cultivars by the deliberate crossing of plants, followed by the selection of the best resulting progeny over several generations.
Cultivar	A homozygous wheat line that has been selectively bred and is cultivated.
Genetic mapping	A method to delimit the position of a trait or gene within the genome. This is generally achieved using genetic or phenotypic markers in conjunction with mapping populations segregating for the trait or gene of interest.
Haplotype	A co‐inherited block of DNA‐containing sequence polymorphisms that in wheat often spans several genes.
Homoeolog	The chromosomes in polyploid species that are derived from different ancestral species (Figure 1) and cannot pair and recombine during meiosis. The genes within these non‐recombining chromosomes are referred to as homoeologs: in wheat there are three homoeologs (A, B and D) for most genes.
Landrace	A domesticated and locally adapted wheat line that is typically grown from farmer‐saved seed and has not been modified through a breeding programme.
Ortholog	Genes in different species that evolved from a common ancestral gene.
Pangenome	The entire spectrum of genetic variation within a species, including genes and other variation found only in a subset of cultivars.
Phenomics	The high‐throughput study of phenotypes.
Phenotype	The physical characteristics of an organism.
Polyploid	An organism with more than two sets of homologous (pairing) chromosomes.
Qualitative trait	A categorical characteristic, such as the number of lateral roots, frequently determined by a single genetic locus.
Quantitative trait	A continuous characteristic, such as yield or grain size, frequently determined by multiple small‐effect genetic loci.
SNP	A single nucleotide polymorphism (SNP) is a variant in a single nucleotide base of DNA, such as a guanine to adenosine (G to A) change.
Wild relative	Plant species that are closely related to a crop species, but are not themselves domesticated or cultivated.

The objective of this review is to discuss how these latest developments can be applied to understand the biology of agronomic traits in polyploid wheat. We describe the new resources and technological developments in the field, and discuss how they can be integrated to improve gene discovery and validation in wheat. We argue that an improved mechanistic understanding of agronomically relevant traits is required for the most efficient deployment of induced and natural variation into the field. This is especially relevant in the polyploid context, where genetic redundancy acts to conceal phenotypic variation. We hope that this review will encourage a wide range of scientists to undertake research for trait discovery in wheat and to further understand biological mechanisms in this fascinating polyploid species. This is an urgent task for achieving global food and nutritional security given the importance of wheat in human diets.

## Loss of Diversity During Domestication and Breeding

**Figure 1:**
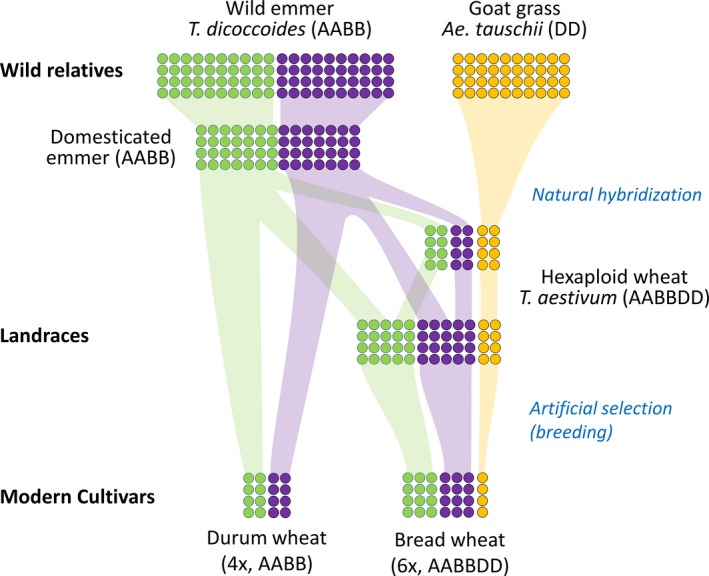
Polyploid wheat genetic diversity. Genetic diversity in wild relatives was lost through the initial domestication of the tetraploid wild emmer and the subsequent natural hybridization that gave rise to the first hexaploid wheat (~10 000 years ago). Inter‐breeding of hexaploid and emmer wheat reintroduced genetic diversity to the A and B genomes of hexaploid wheat (shown by shaded bars) that is present in landraces, whereas the wild progenitor of the D genome (goat grass) was reproductively isolated due to the difference in ploidy levels. Modern‐day cultivars have further reduced genetic diversity due to the bottlenecks imposed by artificial selection (breeding). Circles represent genetic diversity from the A (green), B (purple) and D (orange) genomes. Shaded bars show flow of genetic diversity. Time advances from top to bottom.

On average, each person consumes over 40 wheat plants each day (Data S1). The majority of this consumption (95%) is in the form of bread wheat (*Triticum aestivum*; AABBDD genomes), which is a hexaploid species and most commonly used for bread‐making and biscuit flours. A smaller proportion (5%) is accounted for by durum wheat (*Triticum durum*; AABB genomes), which is a tetraploid species consumed as bulgur and also used to make pasta and couscous. Although distinct species, both share wild tetraploid emmer wheat (*Triticum dicoccoides*, AABB genome) as their common ancestor (Figure 1). While bread wheat arose by the hybridization of domesticated emmer wheat with diploid goat grass (*Aegilops tauschii*, DD genome), pasta wheat arose directly from emmer wheat following two domestication events (Dubcovsky and Dvorak, 2007).

This evolutionary history, in a polyploid context, has multiple consequences that are important to understand trait biology in wheat (Figure 1). Both modern‐day pasta and bread wheat have reduced genetic variation (less than 30%) compared with their wild relatives. This is to an extent due to the domestication bottleneck and selection pressure during breeding (Gaut *et al*., 2018). Even so, this genetic diversity is lower than that found in equivalent diploid cereals, such as maize and millet, which retain over 60% of the genetic diversity from wild relatives (Wright *et al*., 2005). The loss of diversity in wheat is mostly due to the reproductive isolation between closely related species of different ploidy levels, although this loss is not equivalent for all genomes. For instance, the A and B genomes of modern bread wheat retain about 30% of the genetic diversity present in the wild emmer progenitor (Haudry *et al*., 2007), whereas the D genome captures less than 10% of the genetic diversity found in goat grass (Halloran *et al*., 2008). This is because inter‐breeding can occur between wild tetraploid emmer (AABB) and hexaploid wheat, whereas diploid goat grass (DD) and species of higher ploidy levels (such as tetraploid and hexaploid wheat) are reproductively isolated (Dvorak *et al*., 2006; Sutherland and Galloway, 2016; Figure 1). This reduced diversity in modern wheat argues strongly for the use of induced and natural variation to provide novel genetic variation, which has been hitherto unexplored in modern breeding programmes (Winfield *et al*., 2017).

## Homoeologs and Buffering

**Figure 2:**
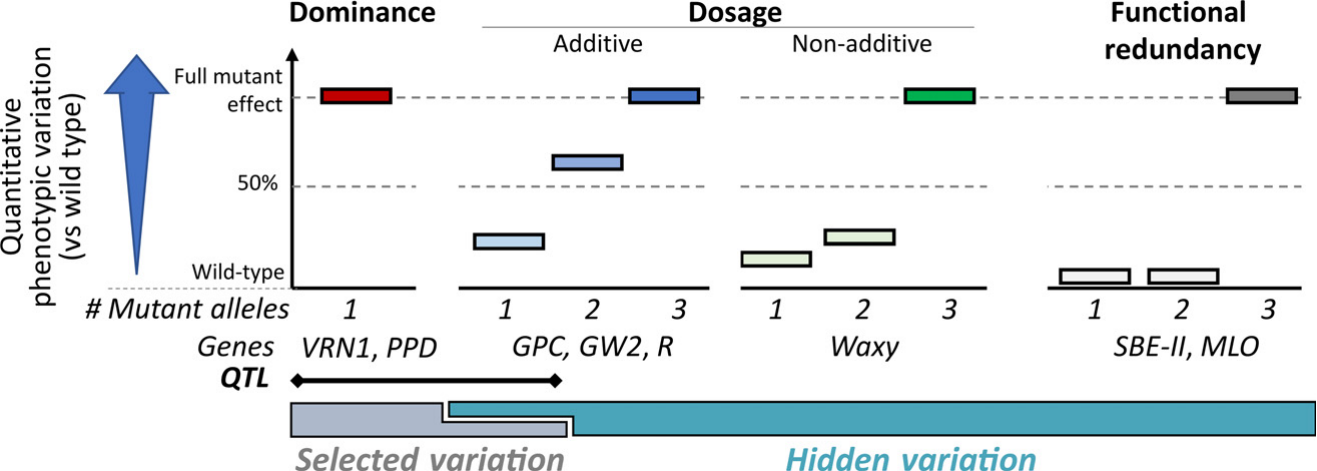
Effects of polyploidy on phenotypic variation. Mutations in a single homoeolog can lead to gain‐of‐function alleles that dominate over the other gene copies (dominance). This variation has been strongly selected upon and includes quantitative trait loci (QTL). Loci displaying dosage effects and functional redundancy are more difficult to select given that changes in one homoeolog lead to subtle or no phenotypic effects. However, combining mutations in multiple homoeologs can uncover an expanded phenotypic spectrum. This constitutes hidden variation of agronomic significance.

The A, B and D genomes have a set of complementary genes (referred to as homoeologs, Box 1) that share between 95 and 98% sequence identity across coding regions [International Wheat Genome Sequencing Consortium (IWGSC) *et al*., 2018]. As a result, many wheat genes are present as two or three functional homoeologous copies (Borrill *et al*., 2015). This affects the phenotypic consequences of variation at a single locus, as this can be masked by redundant copies on the other homoeologous genomes. This is especially relevant for quantitative traits where the genetic complexity of the phenotype is affected not only by the multiple genes that control the trait, but also by the multiple homoeologs of each of those genes. This twofold complexity makes the study of quantitative traits particularly challenging in polyploid species. The main effects of polyploidy on phenotypes are described below and represented in Figure 2.

### Dominance

Mutations in a single homoeolog lead to a gain‐of‐function allele that effectively dominates over the other homoeologous copies. Examples include genes with natural variation in non‐coding regions such as vernalisation (*VRN1*; Yan *et al*., 2003) and photoperiod response (PPD; Wilhelm *et al*., 2009).

### Dosage effect

Changes in one homoeolog lead to subtle phenotypic effects, which are amplified by combining variation in additional gene copies. This dosage effect can be: (i) additive where mutations in increasing numbers of copies lead to proportionally more extreme phenotypes; or (ii) non‐additive whereby single or double mutants have subtle phenotypes that become more extreme when combined as triple mutants. Additive examples include genes controlling grain protein content (GPC; Avni *et al*., 2014), grain size (*GW2*; Wang *et al*., 2018b) and red pericarp colour (*R*; Himi *et al*., 2011), whereas non‐additive genes include *Waxy* controlling amylopectin content (Kim *et al*., 2003).

### Functional redundancy

Mutations in one or two homoeologs of the gene lead to phenotypes that are indistinguishable from wild‐type plants with three functional versions. Only when all three copies are manipulated simultaneously is there a significant difference in phenotype. Examples include genes controlling important quality traits such as amylose content (*SBE‐II*; Slade *et al*., 2012) and recessive disease resistance (MLO; Acevedo‐Garcia *et al*., 2016).

During domestication and breeding, humans have selected most frequently for dominant gain‐of‐function mutations given their strong, easily identifiable phenotypic effects. These include genes with major effects on phenology and adaptation traits as described above, as well as most disease resistance genes (Uauy *et al*., 2017). In contrast, the effects of recessive loss‐of‐function mutations in a single homoeolog are frequently masked by functional redundancy and dosage effects (Figure 2). Hence, phenotypic variation conferred by loss‐of‐function mutations in a single homoeolog are almost indistinguishable from wild‐type plants. This makes their identification difficult, and consequently these loci are seldom detected. In some cases, the variation caused by the loss of a single homoeolog is sufficient to allow their detection as quantitative trait loci (QTL), although the molecular cloning of genes underlying this subtle variation in polyploid wheat has been extremely difficult to date (e.g. GPC). This also explains why many of the examples outlined above (*SBE‐II*,* MLO*,* Waxy*,* GW2*, etc.) were not identified in wheat through phenotypic variation, but rather were characterized in wheat as candidate genes based on knowledge from diploid model species. This leads us to conclude that the wheat genome holds a huge potential of functional variation that has remained hidden and underexploited to date. We argue that gene discovery in wheat is especially relevant to fully exploit this hidden variation as it enables the combination of allelic variation across homoeologs, which in many cases is required to expand the phenotypic spectrum. Identification of causal loci also allows a deeper understanding of allelic variation enabling its fine‐tuning to modulate the plant's phenotype in different environments (for review, see Borrill *et al*., 2015).

## Laying the Foundations for Understanding Genetic Diversity

### Genome assemblies

Attempts to sequence the wheat genome have been hindered by its large size (16 Gb), polyploid nature and large proportion of repetitive sequences (> 85%). Despite this, since 2014 several genome assemblies have been produced for the reference landrace Chinese Spring, including the International Wheat Genome Sequencing Consortium (IWGSC) Chinese Spring Survey Sequence [International Wheat Genome Sequencing Consortium, 2014] and the TGACv1 assembly (Clavijo *et al*., 2017). These two assemblies had good coverage of genic regions, but suffered from fragmentation principally in repetitive regions. More recently, the use of long read technology enabled the production of a more contiguous sequence, the Triticum 3.0 reference (Zimin *et al*., 2017), which assembled in total 15.3 Gb. However, this genome was not annotated with gene models, which restricts its use for gene function studies.

Recently, the IWGSC RefSeqv1.0 genome was released [International Wheat Genome Sequencing Consortium (IWGSC) *et al*., 2018], providing an annotated chromosome‐level assembly for hexaploid wheat. RefSeqv1.0 has a super‐scaffold N50 of 22.8 Mb and a total assembly size of 14.5 Gb, representing 94% of the whole genome. In total, 107 891 high‐confidence gene models were annotated using Illumina and PacBio reads, providing a stable nomenclature from which to build upon. The availability of these gene annotations and the high sequence contiguity have encouraged the uptake of the RefSeq1.0 genome as a community standard, now available through *Ensembl* Plants (Bolser *et al*., 2017). Multiple datasets have now been incorporated into this common reference, such as the physical position of over 4.7 m genetic markers, over 10 m chemical induced TILLING mutants (Krasileva *et al*., 2017) and over 850 RNA‐Seq datasets (Ramírez‐González *et al*., 2018).

The availability of chromosome‐level assemblies is facilitating a step‐change in wheat genetics, enabling the rapid correlation of genetic and physical maps. Whereas historically gene cloning in wheat has relied almost entirely upon genetic mapping of traits, these new alignments will now accelerate trait discovery by placing genetic markers in a physical context and allowing rapid identification of candidate genes within mapped regions. A limitation, however, is that in some cases the population used for genetic mapping may have a different gene content than the reference sequence cultivar, which could lead to candidate genes being missed. This drawback is being addressed through pangenome efforts.

### Pangenomes

With the completion of a high‐quality reference sequence for the wheat landrace Chinese Spring, projects are now focusing on sequencing different wheat cultivars. This is not only important to understand variation within breeding pools and diversity collections, but also to identify sequences and gene content that may be different between the single reference accession and other cultivars used in mapping populations.

Initial efforts focused on the diploid and tetraploid progenitors of wheat: high‐quality assemblies for the known A and D genome diploid progenitor species, *Triticum urartu* and *Ae. tauschii*, respectively, alongside the AABB tetraploid progenitor *T. dicoccoides*, were released in the past 24 months (Avni *et al*., 2017; Zhao *et al*., 2017; Ling *et al*., 2018). A high‐quality reference genome for tetraploid durum wheat, based on the cultivar Svevo, is also available (https://www.interomics.eu/durum-wheat-genome). Concurrently, the 10+ Wheat Genomes project was launched with the aim to provide high‐quality assemblies for cultivars valuable for both research and breeding (www.10wheatgenomes.com). The project aims to sequence 18 different bread wheat cultivars from around the world, some of which are already accessible via online BLAST servers (https://wheatis.tgac.ac.uk/grassroots-portal/blast and http://webblast.ipk-gatersleben.de/wheat_ten_genomes/). The availability of highly contiguous reference genomes for multiple cultivars of relevance in elite wheat breeding programmes will be particularly important to identify structural variants and copy number variation. This type of variation is known to affect traits of agronomic relevance (Gaut *et al*., 2018), yet is difficult to detect with traditional genotyping platforms.

## Genotyping

Alongside the advances in genomic resources, there is a need to rapidly and accurately assess the variation in specific germplasm. The use of ‘SNP‐CHIPs’, arrays of thousands of cultivar SNPs (single nucleotide polymorphisms), has rapidly increased the amount and accessibility of genotype data for specific cultivars of interest (for review, see Uauy, 2017). As sequencing methods and imputation methods improve (Wang *et al*., 2018a), low coverage (skim) sequencing will soon be a viable option in polyploid wheat (Golicz *et al*., 2015). Alongside the large‐scale SNP datasets available at CerealsDB (Wilkinson *et al*., 2016) and *Ensembl* Plants (Bolser *et al*., 2017), advances in polymerase chain reaction (PCR) genotyping markers have facilitated moves into marker‐assisted selection in breeding programs. In particular, the use of the KASP (Kompetitive allele‐specific PCR) technology has provided a sensitive method for bespoke markers within a polyploid context (Ramirez‐Gonzalez *et al*., 2015). Pre‐designed KASP markers are available from CerealsDB and MASWheat for agronomically relevant SNPs such as *GW2*, a grain size gene that is being used in breeding programs (Simmonds *et al*., 2016). These genotyping methods have demonstrated that the polyploid nature of wheat need not be a barrier to obtaining high‐quality, high‐throughput genetic variation information.

## Use of Natural Variation for Trait Discovery

The evolutionary history of polyploid wheat offers several potential sources of genetic variation that can be accessed with different degrees of ease. As discussed above, modern polyploid wheat cultivars have lower genetic variation incorporated from their wild relatives than other major cereal crops such as maize and millet. This lack of diversity is exacerbated for the D genome of bread wheat as only a few *Ae. tauschii* accessions (D genome progenitor) are thought to have been introduced into hexaploid wheat during the natural hybridization process. Thus, efforts have focused on reintroducing variation from *Ae. tauschii* through the re‐synthesis of bread wheat. This is achieved by crossing a tetraploid wheat with a diploid *Ae. tauschii* accession, giving rise to a synthetic hexaploid wheat (SHW; Dreisigacker *et al*., 2008). These SHWs have been shown to be valuable sources of resistance to abiotic and biotic stress (Cossani and Reynolds, 2015). Importantly the introduced D genome from the SHW can freely recombine with modern hexaploid wheat and thus traits can be easily transferred into modern cultivars.

Similar efforts have been made to introduce variation from both evolutionarily near and distant wild wheat relatives (King *et al*., 2016), although these strategies require specialized genetic stocks to allow the transfer of wild wheat chromosome segments. These segments do not freely recombine, therefore, in addition to the beneficial gene, large DNA fragments from the wild relative are introduced often with negative consequences (linkage drag). The use of *ZIP4* mutants can be used to circumvent some of these limitations (Rey *et al*., 2017). In an era of gene‐editing and in which most wild wheat relatives are sequenced (Avni *et al*., 2017; Zhao *et al*., 2017; Ling *et al*., 2018), it may be more efficient to identify genetic variation of interest for a given trait through studies directly in the wild relatives (Golan *et al*., 2015). This genetic variation can then be reproduced or inserted into polyploid wheat via gene‐editing, circumventing the technically difficult introgression process and potential linkage drag. Initial efforts in this direction are already proving useful. Arora and colleagues phenotyped 174 *Ae. tauschii* accessions with several isolates of the stem rust pathogen and sequenced the disease resistance genes from these accessions (Arora *et al*., 2018). Performing a k‐mer‐based association approach called AgRenSeq, they cloned four dominant stem rust resistance genes within months. This knowledge can now be used to introduce the beneficial alleles via transformation, mine for these alleles within modern hexaploid cultivars, or to modify modern sequences via gene‐editing.

An additional important source of accessible genetic variation is found in landraces, locally adapted wheat lines that were grown as farmer‐saved seed and that have not been modified through modern breeding techniques. These landraces constitute reservoirs of genetic diversity that can be directly cross‐bred into modern cultivars. There are thousands of these landrace accessions available; however, many of them are heterogeneous, often duplicated seed stocks (Singh *et al*., 2018), which remain confined to germplasm repositories. Of special note is the Watkins landrace collection (Wingen *et al*., 2014) encompassing over 1000 landraces collected in the 1920s and 1930s from a wide geographic distribution. This collection has been purified by single‐seed descent from which many genomic and genetic resources have been developed. Genotyping of this collection revealed extensive novel genetic (Winfield *et al*., 2017) and epigenetic (Gardiner *et al*., 2018b) diversity, which was absent from modern cultivars. A core set of 107 accessions was used to generate nested association mapping populations (Wingen *et al*., 2017), all of which were genotyped, have genetic maps available, and are free to access (http://wisplandracepillar.jic.ac.uk/).

Modern cultivars also provide useful genetic variation for trait discovery despite this variation being overall reduced with respect to landraces and wild relatives (Figure 1). Much of this variation is studied through genetic populations composed of cultivars that differ for the trait(s) of interest. These include bi‐parental populations between two cultivars (Saintenac *et al*., 2018), multi‐parent advanced generation inter‐cross (MAGIC) populations composed of between 4 and 16 cultivars (Huang *et al*., 2012; Mackay *et al*., 2014; Milner *et al*., 2015; Dixon *et al*., 2018), nested association mapping panels of multiple cultivars to a common parent (Jordan *et al*., 2018), and association panels of 100 or more cultivars (Sukumaran *et al*., 2015; Liu *et al*., 2017). All of these types of populations are available in wheat and their relative merits are discussed elsewhere (Huang and Han, 2014). Importantly, the coupling of these populations to the new genomic and validations tools (discussed below) have provided an integrated workflow for gene discovery in wheat, exemplified by the Triticeae Toolbox from the TCAP project (Blake *et al*., 2016).

The availability of phenotypic data for these vast collections of genotyped landraces, cultivars and wild relatives opens the possibility to assign a breeding value to each polymorphism. This approach enables the implementation of statistical models to define the best combinations of SNPs to maximize the breeding value of progeny from a genetic cross. This genomic selection approach, first implemented in cattle, is used in many crops including wheat (Bassi *et al*., 2016). This approach, however, does not aim to perform trait discovery as it is agnostic of the functional effect of the polymorphism being selected. While effective for highly heritable traits and major effect loci, many of which are already known, this approach is best suited for standing variation within modern cultivars and makes it difficult to incorporate diversity from non‐adapted cultivars or accessions.

## Moving From SNPS to Haplotypes in Breeding

Breeders are constantly shuffling SNPs by making specific crosses and exploiting the subsequent recombination that occurs during meiosis. These SNPs, however, are not inherited independently, but rather as haplotypes; blocks of polymorphisms with a defined order and which in many cases extend across several genes. As genome assemblies have improved in wheat, we are now able to anchor the millions of SNPs previously identified onto the full reference sequence. With these data, it becomes clear that, in the absence of the causal polymorphism *per se*, it is important to recognize the haplotype as the unit of genetic information that underlies traits, rather than a closely associated bi‐allelic SNP. The case study of the wheat pre‐harvest sprouting resistance gene *TaMKK3* provides a telling example to this effect (Barrero *et al*., 2015; Torada *et al*., 2016; Shorinola *et al*., 2017). Thus, when making crosses, breeders are shuffling haplotypes to generate new combinations of these blocks of DNA.

**Figure 3:**
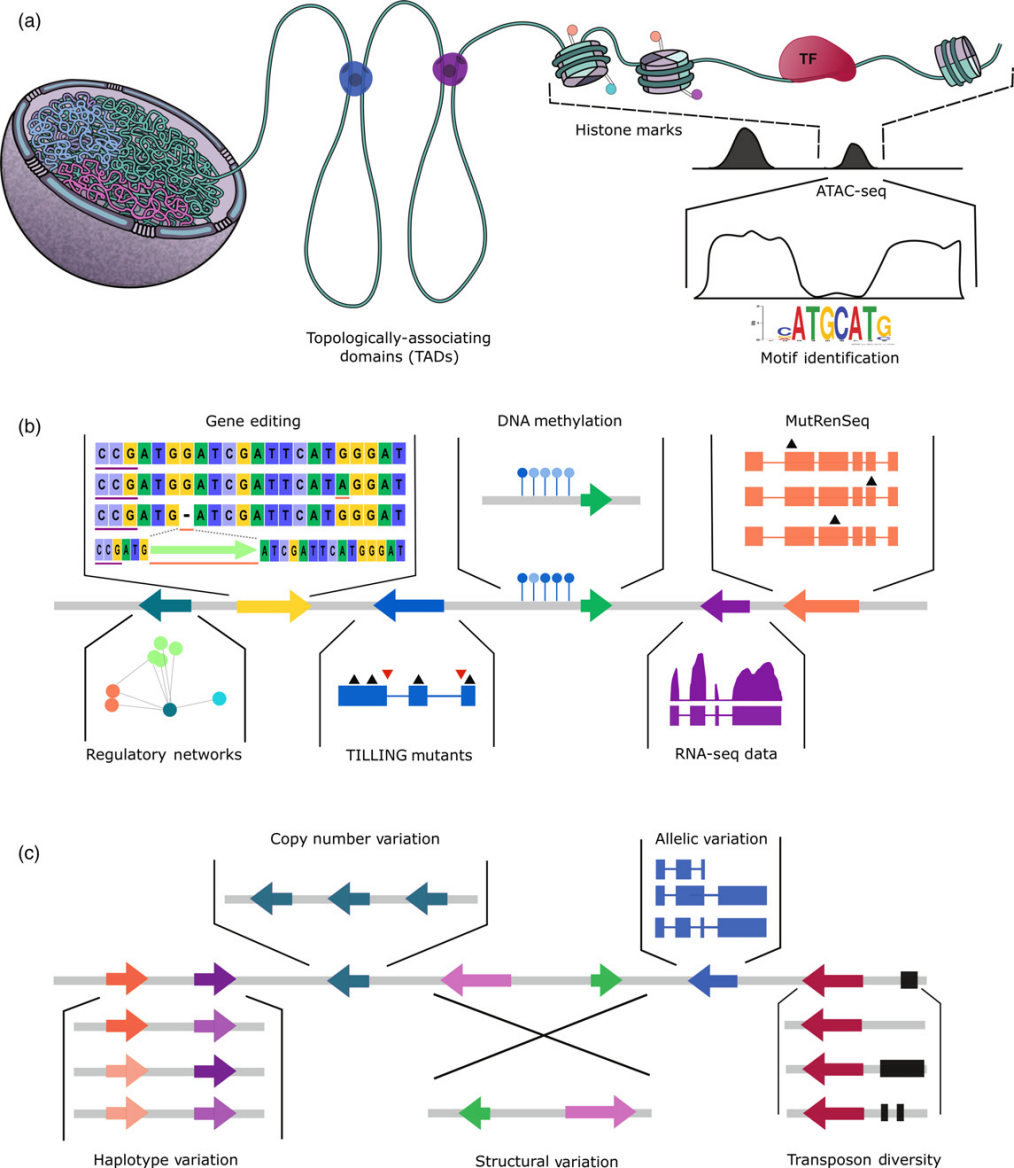
The future of wheat genomics. (a) A new frontier for wheat genomics research will focus on the effects of chromatin structural variation on gene expression regulation. Techniques such as Hi‐C can be used to identify topologically associating domains (TADs), chromatin regions that preferentially interact with each other. Such TADs can affect gene regulation by, for example, bringing distal regulatory elements such as enhancers into proximity with the regulated promoter. Regions of open chromatin can be identified by techniques such as ATAC‐seq, the data from which can then be parsed to identify putative binding sites for regulatory proteins such as transcription factors (TFs). Alongside investigation of chromatin conformation, studies into histone modifications are also necessary to further investigate the impact of epigenetic control on trait variation. (b) The new reference sequence has facilitated the development of many resources and techniques for studying gene function and regulation. Induced variation has been coupled with exome capture and targeted gene‐family enrichment sequencing to study gene function. The *in silico* TILLING database identifies mutations in genes of interest, while techniques like MutRenSeq leverage targeted sequencing to rapidly clone *R*‐genes. New advances in gene‐editing technologies facilitate specific genetic changes, ranging from small indels and specific base‐pair changes through to complete gene insertions. Expression data in wheat are now easily accessible through sites such as www.wheat-expression.com and the eFP browser. Gene networks based on RNA‐Seq data will facilitate our understanding of gene regulatory pathways in wheat. The improved genome sequence will also aid the study of epigenetic variation in wheat, particularly DNA methylation in coding and non‐coding regions. (c) The sequencing of the wheat genome has started to provide insights into genetic variation between cultivars. Haplotype variation within genes is already available and will become increasingly informative as haplotypes extend across larger intervals and recombination blocks are defined. Gene annotations across wheat cultivars and wild relatives will provide insight into allelic variation across the wheat pangenome. Comparisons of transposon diversity, structural and copy number variation between cultivars will also become increasingly possible with improved genome assemblies. These multiple layers of variation data will facilitate hypothesis generation and gene discovery in wheat. See text for references.

The identification of haplotypes allows us to investigate and improve traits both through a retrospective and a prospective approach (Bevan *et al*., 2017). The retrospective approach takes advantage of the rich history of wheat breeding that has very well‐defined pedigrees for most cultivars, many of which can be traced back to landraces. Given that recombination is not evenly distributed across the length of the chromosomes (Akhunov *et al*., 2003), many haplotypes in elite cultivars today will have remained intact as a unit during this breeding process (albeit shuffled and assembled in different combinations with other haplotypes). This allows us to retrospectively evaluate which haplotypes have been selected and enriched for in certain environments and which haplotypes have been lost, potentially due to deleterious effects. Thus, we can benefit from years of available phenotypic data and selection to assess haplotype × environment interactions. This retrospective approach has highlighted, for example, how strong selection for flowering time has eliminated haplotypes for increased root biomass, a desirable trait (Voss‐Fels *et al*., 2016). We can now use this knowledge to break these negative correlations by purposely generating haplotypes that combine both beneficial traits. This will be facilitated by ongoing studies into the genetic basis of recombination in wheat, which could provide the knowledge to manipulate recombination rate distribution across chromosomes (Jordan *et al*., 2018). With clearly defined haplotypes, we can also leverage the multiple layers of genomic information (e.g. networks, RNA‐Seq; Figure 3) to help identify the causal variant underlying the trait of interest.

By understanding the haplotypes available within elite cultivars, we can also use a prospective approach to mine landrace and wild relative collections for novel haplotypes. This approach could entail mining for haplotypes that break negative correlations across specific low‐recombinogenic regions (Winfield *et al*., 2015), or alternatively mining for novel haplotypes across QTL loci associated with a known agronomic trait (Muterko *et al*., 2015). This will become more commonplace as large diversity collections are genotyped at high‐density and haplotype imputation becomes more robust (Jordan *et al*., 2015). In this manner, rather than phenotyping a large diversity collection for many traits, the phenotypic value of different haplotype blocks across a specific interval could be examined in further detail. The Watkins nested association mapping populations offer a valuable resource to this effect as the landrace haplotypes can be directly evaluated within available mapping populations (Wingen *et al*., 2017). As breeding programs increasingly look to combine genotypic and phenotypic data, the availability of haplotype data for wild relatives, landrace collections and elite wheat cultivars with their associated phenotypic value has the potential to shape the way in which breeding programs are structured.

## New Strategies Accelerate Gene Discovery in Wheat

With the genomic resources developed recently for wheat, many new techniques for gene discovery have been developed. One area has focused on rapid cloning of disease resistance (*R*−) genes by using targeted genome sequencing approaches (Keller *et al*., 2016; Periyannan, 2017). MutRenSeq takes advantage of significantly improved *R*‐gene annotations to use an *R*‐gene enriched capture method to sequence the majority of *R*‐genes in a given plant (Jupe *et al*., 2013; Steuernagel *et al*., 2016; Figure 3). Combining *R*‐gene enrichment sequencing with screening of mutagenized populations for dominant disease resistance phenotypes has allowed easier and more rapid cloning of *R*‐genes than previously possible via traditional genetic mapping (Marchal *et al*., 2018). As described above, a variation on the technique, AgRenSeq, uses the enrichment strategy to identify variation in the *R*‐genes of wild relatives (Arora *et al*., 2018).

While the whole genome sequence is now available, it remains cost and time‐prohibitive in most cases to fully re‐sequence lines for gene discovery. Instead, integrating known genetic mapping with new genomic approaches can accelerate trait discovery. Exome‐capture sequencing has been used to identify variation in the coding region of the wheat genome (Saintenac *et al*., 2011) and can be used to identify candidate genes, often in combination with bulk‐segregant analysis (Gardiner *et al*., 2016; Mo *et al*., 2018). SNPs identified from the exome capture can be used to both develop new markers for genetic mapping and to identify mutations in putative candidate genes. However, this technique is limited by the quality of the capture probes used. Many current capture arrays were designed based on the gene models from the 2014 genome, and as such are missing or mis‐representing various genes (Brinton *et al*., 2018). Efforts are underway to develop new capture arrays based on the improved annotations (Gardiner *et al*., 2018a).

Alternative techniques aim to reduce the size of the genome by flow‐sorting and sequencing single chromosomes (Giorgi *et al*., 2013). One approach, MutChromSeq (Sánchez‐Martín *et al*., 2016), requires a mutation‐induced phenotype that can be readily screened and has been mapped to a specific chromosome. The target chromosomes of independent mutants are then flow‐sorted and sequenced before being compared to identify candidate genes. A second approach, TACCA (Thind *et al*., 2017), relies on knowledge of a specific target interval (based on flanking markers) identified by traditional genetic mapping, and as such is amenable to both qualitative and quantitative traits (Box 1). The aim of this method is to build, in one step, long‐range contiguous assemblies that completely span the target interval, thereby eliminating the need for BAC clones. Flow‐sorted chromosomes from the cultivar of interest are sequenced and assembled using the Chicago method. If the resulting long‐range assemblies are contiguous enough to contain the flanking genetic markers, then identification of the causal variation can be rapid. These approaches allow for the unbiased discovery of variation beyond the genic variation identified with exome capture, and also aid the identification of causal variants in cultivars that differ from the established reference cultivars. Looking forward, CRISPR‐Cas9 may be used to cut at flanking marker sites to generate a relatively short piece of genomic DNA that can be separated from other genomic DNA by size selection before sequencing. This approach, which has been carried out using human DNA (Nachmanson *et al*., 2018), albeit with relatively closely spaced cut sites, would avoid the requirement for chromosome flow‐sorting that is technically challenging and is only routinely carried out in a few laboratories worldwide.

## Hypothesis Generation in Wheat

### Orthology

One of the more tangible results of the improved genome assemblies and gene annotations is the increased ease with which knowledge from other species can be transferred into wheat (Adamski *et al*., 2018). High‐quality gene annotations based on the new assemblies are now included in platforms such as *Ensembl*Plants, which identify putative orthologous genes in other species. This knowledge base facilitates the application of reverse genetic approaches to wheat using orthologs of genes identified in other species. Once identified, these genes can be tested for function in wheat using sequenced mutant populations (Krasileva *et al*., 2017), as will be discussed in more detail below. An important caveat to any reverse genetics approach is that known function cannot be presumed to be conserved between species, particularly when moving from a dicot species such as Arabidopsis into wheat. Comparative studies on the flowering time pathways between Arabidopsis and cereals show that while some core pathways have been conserved across species, there is lineage‐specific divergence (Higgins *et al*., 2010). However, the candidate gene strategy has been successful in identifying putative genes in wheat, particularly from rice orthologs, which have gone on to be characterized and confirmed, such as *GW2* (Simmonds *et al*., 2016; Wang *et al*., 2018b; Zhang *et al*., 2018) and other regulators of grain size (Li and Yang, 2017).

Developments of resources in other Triticeae species, especially barley, may also enable the rapid transfer of knowledge amongst this phylogenetic clade. This approach has been successful for genes relating to, for example, flowering time (Turner *et al*., 2005; Beales *et al*., 2007), cuticular wax biosynthesis (Hen‐Avivi *et al*., 2016; Schneider *et al*., 2016) and pre‐harvest sprouting (Nakamura *et al*., 2016; Torada *et al*., 2016). Likewise, genes identified in tetraploid wheat can be rapidly evaluated in hexaploid wheat as most genes identified in emmer and durum wheat perform equivalent roles in hexaploid wheat (Uauy *et al*., 2005, 2006).

### Expression

The decreasing cost of RNA‐Seq and improved wheat genome sequences has enabled a rapid increase in large‐scale gene expression analyses in wheat (Figure 3). Software, such as the pseudoaligner kallisto (Bray *et al*., 2016), can readily distinguish between sequences from highly similar homoeologs (Borrill *et al*., 2016), allowing homoeolog‐specific gene expression analyses. These analyses have been used to understand the pathways regulating varied developmental and stress‐related traits (Kugler *et al*., 2013; Brinton *et al*., 2018), to understand the roles of individual genes (Pearce *et al*., 2014, 2016), and to compare response between stress treatments (Zhang *et al*., 2014; Liu *et al*., 2015). These examples highlight the many ways in which RNA‐Seq datasets can be used to shed light on the regulatory pathways involved in different traits of interest and to identify candidate genes for hypothesis generation.

To maximize the utility of these data, many of these studies have been incorporated into gene expression atlases. The expVIP gene expression atlas contains over 900 RNA‐Seq samples from a range of diverse tissues, developmental stages, stresses (abiotic and disease) and cultivars (Borrill *et al*., 2016; Ramírez‐González *et al*., 2018). Metadata categorizing the samples allows for comparisons between different studies, and enables sorting and filtering of relevant samples on the web interface (www.wheat-expression.com). A highly detailed developmental timecourse of the spring wheat cultivar Azhurnaya (Ramírez‐González *et al*., 2018) is available both through expVIP and a dedicated eFP browser (http://bar.utoronto.ca/efp_wheat/cgi-bin/efpWeb.cgi; Winter *et al*., 2007). This pictographic display facilitates rapid assessment of the expression pattern of a gene across 70 different tissues and developmental stages.

**Figure 4:**
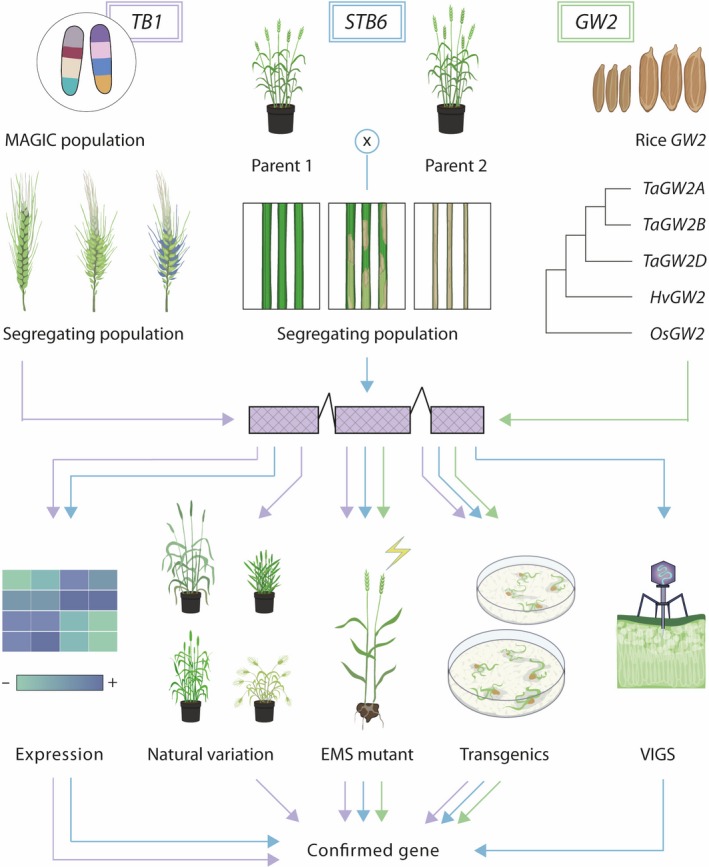
Integrated workflow for gene discovery in wheat. The development of genetic and genomic resources has accelerated candidate gene discovery in wheat. For example, multi‐parent advanced generation inter‐cross (MAGIC) populations were used to identify the wheat *Teosinte Branched 1* (*TB1*) homolog as a major regulator of inflorescence architecture and development (Dixon *et al*., 2018); the use of mapping populations and full genome sequence of the landrace reference and cultivars facilitated the identification of a candidate for the *Zymoseptoria tritici* resistance gene *STB6* (Saintenac *et al*., 2018); homology with rice enabled identification of *Grain Width 2* (*GW2*) (Wang *et al*., 2018b). Starting from a defined candidate gene (purple gene model in centre), there are multiple strategies for gene validation available in wheat. These include expression datasets, natural variation in cultivars and landraces, *in silico* EMS‐mutants, multiple transgenic validations including gene editing, and transient transformation systems such as virus‐induced gene silencing (VIGS). Studies have combined these approaches to confirm the gene underlying the trait of agronomic interest. Arrows denote the paths used to identify and validate *TB1* (purple), *STB6* (blue) and *GW2* (green).

These readily accessible gene expression data enable hypothesis generation based on expression patterns (Figure 4). Comparisons of expression data between orthologous genes can provide insight into shared or divergent functions between species. Expression data can also be a useful source of supporting information to narrow down candidate genes identified through conventional QTL mapping or functional genomic approaches using natural and induced variation.

### Networks

Although knowledge about individual gene's expression patterns is very useful, it is also important to consider how genes act together in networks to regulate phenotypes. In model species such as Arabidopsis, network approaches have been successful in identifying candidate genes regulating specific processes (Bassel *et al*., 2012), and recent comparative work has uncovered conserved gene networks in root tips across monocots and dicots (Maher *et al*., 2018). Network‐enabled approaches are likely to find broad applications in wheat research, now that the necessary genomic resources are available (Figure 3).

Early work by WheatNet (https://www.inetbio.org/wheatnet) generated a wheat network using 20 genomics datasets, although only one of these was derived from wheat‐specific data (Lee *et al*., 2017). Knetminer (http://knetminer.rothamsted.ac.uk) also presents a wheat network, which until recently was based mainly on information derived from orthology (Hassani‐Pak *et al*., 2016). The use of co‐expression studies has been relatively limited, although genes and pathways associated with fusarium head blight resistance (Kugler *et al*., 2013) and spike architecture (Wang *et al*., 2017) have been uncovered. Recently, a set of co‐expression networks was developed using publicly available and newly generated RNA‐Seq samples for specific tissue types (leaf, root, grain and spike) and abiotic or disease stress conditions (Ramírez‐González *et al*., 2018). The development of an integrated network for all 850 RNA‐Seq samples provides an entry point into understanding the general co‐expression patterns of genes across a wide range of tissues [International Wheat Genome Sequencing Consortium (IWGSC) *et al*., 2018], and has been incorporated into Knetminer. Expression data have also been leveraged to generate a network that predicts downstream targets of transcription factors (Ramírez‐González *et al*., 2018), also available in Knetminer. A powerful approach in the future will be to layer wheat‐specific information on top of information from model species to identify candidate genes and groups of genes regulating specific traits of interest.

An important question to consider when working with a polyploid species is whether the homoeologous copies of genes are functionally redundant. These co‐expression networks have started to address this question and show that whilst many homoeologs have the same or similar expression patterns, approximately 30% of homoeologs have divergent expression patterns from each other (Ramírez‐González *et al*., 2018). This implies that a significant proportion of wheat genes may already be undergoing the first steps towards sub/neo‐functionalization through changes in their patterns of expression. This information can be used to inform strategies to alter gene function, for example, altering a gene whose homoeolog has a very divergent expression pattern is more likely to cause a phenotypic effect than if the homoeologs had highly similar expression patterns.

## Gene Validation in Polyploid Wheat

As the new genome assemblies increase the potential for gene discovery, the focus now turns to ways in which gene function can be characterized (Figure 4). Exome‐capture followed by next‐generation sequencing has been applied to tetraploid and hexaploid TILLING populations to identify and make available induced variation in gene coding regions (Krasileva *et al*., 2017). This database allows for ‘*in silico* TILLING’ whereby mutations in any gene of interest can quickly and easily be identified. Importantly, while the SNPs were originally called against the 2014 IWGSC reference genome (available at www.wheat-tilling.com and https://dubcovskylab.ucdavis.edu/wheat-tilling), they have recently been remapped to the RefSeqv1.0 gene models and are available on *Ensembl*Plants. The mutagenized populations are particularly useful due to their ease of access, with researchers being able to identify mutations in their gene of interest within minutes. However, because the sequenced mutations will lead to a truncation (premature termination codon or splice junction variant) in only 60% of genes, the use of missense mutations will also be required at times. As a result, screening more than one mutation per gene might be necessary as predicting the functional effects of missense mutations is not always easy *a priori*. Similarly, use of the TILLING lines typically requires multiple iterations of crossing, first to combine mutations across homoeologs of the gene of interest and secondly to reduce background mutations (Krasileva *et al*., 2017; Uauy *et al*., 2017). Typically, for quantitative traits, phenotypes are clearer with successive backcross generations (Simmonds *et al*., 2016). However, some qualitative traits can be studied directly in the initial mutant population (Mo *et al*., 2018; Shorinola *et al*., 2018). A caveat for the use of these populations is that not all genes will be present in the *in silico* database. This is either a result of the genes not being present in the exome‐capture array, or because these genes are not present in the cultivars used to create the populations. In these cases, identifying mutations in the gene(s) of interest would require the screening of the population using conventional TILLING approaches or the development of a new bespoke mutant population using the cultivar with the gene(s) of interest.

Alternative methods for gene functional characterization focus on transgenic techniques. These can be either transient or stable transformation methods, and are able to influence gene expression levels beyond what is possible via mutagenesis. Transient methods benefit from the relative ease and speed of transformation compared with stable integration, with several transient gene expression and silencing systems now available in wheat. The use of virus‐induced gene silencing (VIGS) in wheat and other cereals has been highly successful in characterizing the impact of various genes involved in abiotic and biotic stress response (Cakir *et al*., 2010; Ramegowda *et al*., 2014; Lee *et al*., 2015). These VIGS systems are now well established for use in wheat, and more recently advances in virus‐mediated overexpression have demonstrated the feasibility of their use in wheat for both small and large proteins (Bouton *et al*., 2018; Cheuk and Houde, 2018). Beyond viral expression systems, other possibilities for transient expression in wheat exist. Nanomaterials have recently shown potential for the transient delivery of foreign DNA into wheat leaves and protoplasts (Demirer *et al*., 2018).

Despite their increased cost and lengthier transformation process with respect to transient systems, traditional stable transgenics continue to be used in many contexts to validate and explore the function of genes in wheat (for review, see Borrill *et al*., 2015). A major bottleneck of current wheat transformation systems is that only very few, typically non‐elite and non‐adapted wheat cultivars (such as Fielder or Bobwhite) are transformable at reasonable frequencies (Harwood, 2012), although this is changing (Richardson *et al*., 2014; Ishida *et al*., 2015; Wang *et al*., 2016). Studies in maize have demonstrated that the concurrent expression of transcription factors *Baby boom* and *Wuschel2* increase both transformation efficiency and the range of cultivars that are transformable (Lowe *et al*., 2016). Integration of this system into wheat transformation platforms could allow genotype‐independent transformation, which would accelerate the timeline for integration of transgenic traits into wheat breeding pipelines, while also making it easier to evaluate the effects of the transgene or modification in a locally relevant cultivar.

Genotype‐independent transformation is of particular interest when considering applications for gene editing, which are not regulated as genetically modified organisms in North America, Japan and elsewhere, though they are in Europe. Gene‐editing systems such as TALENs and CRISPR‐Cas9 have now been demonstrated to function in wheat including the production of transgene‐free edited lines (Wang *et al*., 2014; Zhang *et al*., 2016; Rey *et al*., 2018). Beyond the most common use of CRISPR‐Cas9, to induce small deletions that lead to frame‐shifts and knock‐outs, this may involve more complex editing such as targeted gene insertion or specific base editing (for review, see Adli, 2018). In theory, the use of guide‐mediated gene editing could produce triple homoeolog knock‐outs in a single transgenic event, although efficiencies remain low (Zhang *et al*., 2016). As the use of this system becomes more commonplace in wheat, we anticipate that advances in transformation and editing efficiency will follow, increasing the impact of the system. Crucially, the ability to transform elite wheat cultivars would significantly accelerate the rate at which edited alleles could be deployed in farmers’ fields.

## Developments in Phenotyping

Historically, wheat phenotyping for major agronomic and quality traits (e.g. yield, protein content) has required field trials that are labor‐intensive and can only be carried out once per year in many parts of the world. To overcome this bottleneck (Furbank and Tester, 2011), one area of recent growth has been the use of high‐throughput phenotyping systems. Protocols have been developed for controlled environments and field conditions, enabling a wide range of traits to be studied. Large collections of germplasm in wheat have been amassed (see above), but as yet relatively few collections have been screened using high‐throughput phenomics, presenting an opportunity for future research.

Many traits, including growth rates, biomass and abiotic stress responses, have been studied in high‐throughput phenotyping platforms in glasshouse conditions, particularly in specifically constructed facilities with conveyor belts for frequent imaging of plants. Different cameras are required for imaging specific traits: growth rate and biomass can be calculated from standard RGB images (Parent *et al*., 2015), whereas infra‐red imaging has been used to study osmotic stress (Sirault *et al*., 2009). One area of research that has particularly benefited from glasshouse‐based phenomics is the understanding of root architecture. Roots are a particularly difficult tissue to study because access in the field requires excavation and cleaning of the soil through methods such as ‘shovelomics’, which has been applied in maize (Trachsel *et al*., 2011) and recently wheat (York *et al*., 2018). Recently developed higher‐throughput technologies have enabled the phenotyping of root architecture in wheat seedlings using transparent pots (Richard *et al*., 2015) or through paper‐based screening (Atkinson *et al*., 2015). These technologies have now been applied to screen sequenced mutation populations to identify mutants with altered root architecture (Shorinola *et al*., 2018). Adult plant roots pose a greater challenge due to their larger size, but developments in root tracking approaches (Mairhofer *et al*., 2015; Pfeifer *et al*., 2015) are increasing the rate at which high‐resolution root systems can be imaged through magnetic resonance imaging and X‐ray microcomputer tomography.

Controlled environment conditions enable the rapid screening of seedling stage plants; however, the strength of the correlation between seedling physiological traits and those of plants growing in the field may be poor for some traits. For example, a meta‐analysis for specific leaf area, leaf nitrogen concentration and yield across a range of crop species only showed a median correlation of 0.26 between the lab and the field (Poorter *et al*., 2016). However, steps can be taken to narrow this gap, for example by applying abiotic conditions more similar to those found in the field to controlled environment experiments, growing plants in controlled environments in more natural soils, or by planting at densities more comparable to the field (Poorter *et al*., 2016).

Therefore, in parallel with pot‐based approaches in glasshouses, in‐field technologies have been developed. These are usually either a fixed platform with a camera that images a small area of selected plots in the field or based on mobile imaging systems such as manually operated platforms or unmanned aerial vehicles. The specifics of these platforms have been reviewed recently (Araus *et al*., 2018), and each offers a unique set of advantages and disadvantages. These platforms all rely on imaging technologies such as RGB and infra‐red, mentioned previously, as well as hyperspectral imaging, which is particularly popular for in‐field imaging. Hyperspectral imaging uses wavelengths across the electromagnetic spectrum to generate indices such as normalized difference vegetative index to measure canopy coverage (Christopher *et al*., 2016), as well as to calculate models for specific biochemical and physiological traits (Silva‐Perez *et al*., 2018). Hyperspectral imaging can also be used to detect and classify disease and stress symptoms; however, collecting appropriate data and developing analysis pipeline can be a challenge within commercial breeding programmes (Lowe *et al*., 2017). At CIMMYT large‐scale phenotyping for physiological traits has been incorporated into breeding programmes using both high‐throughput imaging and conventional phenotyping methods (Reynolds and Langridge, 2016). The phenotypes obtained through these methods have been used to inform physiological breeding, in which lines with complementary physiological phenotypes are crossed. This approach has successfully bred lines with improved phenotypes for a range of traits, including yield potential, drought and heat stress (Reynolds and Langridge, 2016). Subsequent genetic dissection of the physiological traits in the improved lines can reveal the loci underpinning these phenotypes, which can then be used in marker‐assisted selection across the breeding programme. Combining phenomics with genotypic information is also proving useful, for example to improve genomic selection for quantitative traits such as yield (Crain *et al*., 2018).

Both in‐field and glasshouse‐based phenomics platforms generate huge quantities of image data that requires processing to extract the relevant information. However, in comparison to genomics analyses, pipelines for image processing are far less standardized and represent a major informatics challenge. As these phenomics approaches have become more widely adopted, a number of tools for image processing from small‐scale glasshouse experiments to large hyperspectral field experiments have been developed (Perez‐Sanz *et al*., 2017). Machine learning is a promising approach to accelerate data processing and improve trait analysis (Singh *et al*., 2016), although obtaining high‐quality annotated images will also be essential (Tsaftaris *et al*., 2016). In the future it is likely that standard procedures for analysis will be developed, analogous to standard genomic pipelines such as GATK for variant calling (Mckenna *et al*., 2010).

## Looking Forward

Connecting genes to phenotypes in wheat is challenging, but combining the two is urgent and necessary to accelerate breeding efforts to produce enough food for the world's population. Efforts to develop new, adapted wheat cultivars will require improved understanding of the interactions between genotypes, environment and management practices, in the context of climate change (Challinor *et al*., 2014). Despite these challenges, there are great opportunities to understand wheat biology in the coming years.

One area of wheat biology that is as yet underexploited is the modulation of individual homoeologs to influence traits. As we increase our understanding of the extent to which homoeologs are functionally redundant, new approaches such as epigenetic modification, altering promoter sequences or introducing dominant alleles could be exploited to produce a wider range of phenotypes using the genes that wheat already contains. Many of these alterations could build upon novel gene‐editing technologies. For example, genome‐editing within promoter regions in tomato has expanded the range of fruit sizes, inflorescence branching and plant architecture (Rodríguez‐Leal *et al*., 2017). Use of this technology in wheat could expand the phenotypic diversity available for key genes. Targeted engineering of individual homoeologs might unlock previously hidden phenotypes, while the ability to carry out allele replacement will allow the rapid transfer of desirable alleles into elite breeding material.

The impact of chromatin conformation on phenotypic variation is perhaps even less investigated, yet has the potential to provide substantial insights into the connection between genotype and phenotype. In animals and plants, accessible (open) chromatin has been associated with functional non‐coding sequences. In maize, for example, < 1% of the genome was defined as open chromatin, yet variation within these regions explained 40% of phenotypic variation for many quantitative traits (Rodgers‐Melnick *et al*., 2016), consistent with studies in humans and Arabidopsis (Maurano *et al*., 2012; Sullivan *et al*., 2014). Techniques such as Hi‐C (Belton *et al*., 2012) and ATAC‐seq (Buenrostro *et al*., 2015) can be applied to the wheat genome to characterize chromatin structure and identify putative regulatory motifs (Figure 3a). As 99% of the wheat genome is non‐protein coding, variation in these regions is likely to account for a significant proportion of phenotypic variation. DNA methylation and histone mark variation both within and across cultivars is only beginning to be studied in wheat (Gardiner *et al*., 2018b; Ramírez‐González *et al*., 2018), and is likely to provide a wealth of information on gene regulation for traits of agronomic importance. Natural epigenetic variation is known to impact on agronomically relevant traits such as fruit ripening and plant height, and was found to underpin artificially selected variation for increased energy use efficiency in canola (Springer, 2013; for review, see Gallusci *et al*., 2017, 2013). As wheat genetics moves further towards understanding hidden variation derived from polyploidy, it is now also becoming possible to consider untapped variation in the context of chromatin structure and epigenetic modifications.

Advances in phenomics have enabled us to generate large phenotypic datasets, and now opportunities will arise to understand the links between genotype, environment, agronomy and phenotype at a systematic scale. Machine learning is showing promise in predicting phenotypes in wheat based on genomic data (Grinberg *et al*., 2018), performing better than classical statistical genetics methods. Likewise crop simulation models based on physiology and phenology of wheat have improved in recent years, yet they currently only incorporate genetic effects relating to relatively basic phenotypic traits (e.g. flowering; Chenu *et al*., 2017).

The addition of novel data types such as epigenetics and chromatin conformation may contribute to improved wheat phenotypic prediction. Initial studies reveal epigenetic differences amongst landraces (Gardiner *et al*., 2018b), which are likely to also be observed in elite material. Incorporating diverse data types (genomics, epigenomics and environmental data) may enable more accurate predictions of phenotypes. The use of computation models will also be useful for the development of networks predicting the phenotypic values of haplotype combinations, epi‐alleles and the outcomes of crosses between cultivars. Current genomic and epigenomic approaches rely on whole tissue samples; moving forward, single‐cell approaches (Cao *et al*., 2017) will increase resolution in studies of cell‐specific process. However, we must be cautious that the ability to simultaneously measure many thousands of genes or thousands of phenotypes using new technologies may not necessarily improve our ability to understand the underlying biology. Therefore, we believe that combining these new techniques with targeted studies of specific genes and traits promises to provide insights into the core biological processes regulating agronomically relevant traits in wheat.

In summary, the last decade has brought about a revolution in the resources and approaches used for wheat research and breeding. We now have a wide range of resources that provide opportunities to improve our understanding of wheat gene functions, many of which are easily accessible for researchers moving into wheat from other species (Adamski *et al*., 2018). One of the key challenges moving forwards will be predicting which genes will be most valuable within breeding programmes in diverse and changing environmental conditions.

## Conflict of interest

The authors declare no conflict of interest.

## Supporting information


**Data S1.** Calculations for daily consumption of wheat.
